# Mr. Wilkinson on Cutaneous Diseases

**Published:** 1823-06-01

**Authors:** 


*Remarks on Cutaneous Diseases.** By J. H. WILKINSON. London, pp. 87.

THE task of analysing this little work is rendered less easy than it might have been by the total want of method in the arrangement, and the absence of an index or table of contents. There are, in the whole, four chapters of unequal length. The first of them has not any title—the second is headed "the Prurigo"—the third and longest "Porrigo"—and the last "the Impetigo, &c." In a short preface, the author takes occasion to express his dissatisfaction with the existing "arrangements and treatment" of diseases of the skin; but he does not profess to supply these deficiencies.

It is not, he says, his intention to give a systematic account of these affections, and he appears of opinion subsequently that Dr. Willan has already accomplished, in this point, all that can be desired, as he bestows upon his classification unqualified praise, (pp. 9, 13, 75.) Neither does Mr. Wilkinson propose to offer to our consideration any new remedies, (p. 31;) his chief object being to point out which of those in common use he has found the most efficient, as well as the best mode of applying them. "This," he adds, "is my principal motive for writing these remarks; an additional one is, that this exposure of my simple ways and means has *lately*† appeared to me to be absolutely necessary for the support of the little reputation I had gained; for it has been thought, by many, that I had been in possession of a secret which I was unwilling to divulge; but it will now be perceived, that any little success of which I can boast, has been owing to the combination of the remedies, but still more to the *method* of *applying* them." (P. 32.)

Mr. W. has not any thing new to communicate concern-

---

* We transmitted Mr. Wilkinson's work on Cutaneous Diseases to a gentleman and friend, who has made those diseases a particular, though not an exclusive study. He returned us the following concise analysis of its contents, with only this remark. "It is not my purpose to estimate the merits of this production, but to place the materials for doing so in your hands." Now, in the majority of instances, this is just what we do for, and say to, the public—therefore, to the public it shall go as it came to us.

† It will appear in the sequel, that Mr. W. has employed his two magna remedia, (volatile alkali and unguentum picis cum sulphure,) the one *sixteen* and the other *seventeen years*, with the same happy results. Vide pp. 16 and 73.

ing the anatomy and physiology of the skin, and he passes on at once to the pathology of that organ. He apprehends that writers, in general, have too little regarded the "secretion upon the surface of the true skin, and its power, when altered from its natural state, of producing *all* the different eruptions;" "nor yet how dependent all their varieties are, upon the different states or condition of the skin, upon which such disordered secretion acts," (p. 1.) And he distrusts very much the correctness of "the prevailing opinion that different eruptions are produced by different diseased secretions;" and declares his belief, "that the diseased secretion is always essentially the same; and that the varieties of the eruptions are produced solely by the conformation and constitution of the subject." (P. 2.)

In other words, we understand him to say, that the thirty-eight genera of Dr. Willan's system, and the two afterwards added by Dr. Bateman, (viz. rupia, and a tubercular affection resembling syphilis, described in the fifth volume of the Medico-Chirurgical Transactions) are, with their multifarious varieties produced by one and the same morbid secretion, developing itself according to the idiosyncracy of individuals in the diverse forms of pimples, scales, rashes, blebs, pustules, vesicles, tubercles, and spots.

In imparting to us his methodus medendi, the author begins by acknowledging his obligations to Dr. Peart, (of Butterwick, near Gainsborough.) This physician, rather more than twenty years since, published a small tract,* in which he strongly recommended the volatile alkali as a specific in scarlatina maligna. Mr. W. adopted the use of the medicine; and "consonant," he says, "with my own principle, that an effectual remedy for one genus will, with proper management, cure all the genera of the same order, I administered it in all the following diseases—erysipelas, rubeola, scarlatina, urticaria, roseola, and erythema, with all their varieties; and I am happy to be able to declare that, from that moment to the present, a space of seventeen years, I have not only never lost a patient in the above diseases, but I have never had a case of the kind that has ever appeared dangerous, or that has even given me a moment's uneasiness." (P. 16.) He conceives that erysipelas has been improperly classed in the order bullæ, by Willan and Bateman, and that the inflammatory action attending the disease is more analogous to that which accompanies some of the exanthemata.

It may not be improper to remark, in this place, that Dr. Peart published, at a period subsequent to the appearance

* Practical Information on the Malignant Scarlet Fever and Sore Throat.

of the address alluded to by Mr. W. a *second*, which has evidently escaped that gentleman's notice, for the express purpose of advising the extension of his remedy (the volatile alkali) to the treatment of rubeola, erysipelas, and *erythematous affections in general:* and, at the same time, he states it as his opinion, that these diseases and scarlatina "are branches of the same radix."*

Mr. W. in introducing the subject of porrigo, expresses his conviction that this offensive disorder has become very much more prevalent within the last quarter of a century, and he is inclined to attribute this circumstance to the large number of children sent, during that period, to this country, from the East and West Indies; and by whom, he conceives, the infection has been imported, and transmitted, through the medium of schools, into all parts of the island.

His statement, in regard to the non-contagious nature of the porrigo larvalis, or crusta lactea, concurs with that of Dr. Bateman, in the last edition of his synopsis, and the same fact has been more recently noticed by Dr. Smith, in Dr. Willan's Miscellaneous Works.—(Reviewed in the Medico-Chirurgical Journal, for June last.) But, we believe, Mr. W. is singular in maintaining that the porrigo favosa is not infectious. (P. 54.) In treating of the first-mentioned variety, he takes occasion to combat a notion which, he says, obtains among "some of the most eminent practitioners of the present day," that this eruption depends upon *poorness of blood.* P. 47. In respect to porrigo perforans, he doubts the accuracy of the assertion of Drs. Willan and Bateman, that it chiefly affects adult subjects; and observes that, in his own practice, the proportion of patients affected with this complaint has been as one adult to thirty children. He does not think that this variety of porrigo *commences* with achores, but that these pustules arise during its progress from neglect of cleanliness. In the treatment of porriginous affections, the author relies principally upon the very liberal application of an ointment, which he has likewise found useful in the squamous and some other diseases of the skin, and for the preparation of which he gives the following formula:—

℞. Sulph. sublim.
Picis liquidæ.
Axung. porcin. āā Oss.
Terræ cretos, ℥iv.
Hydrosulph. ammon. ʒij.
M. ft. unguentum.

---

* See Practical Information on the Erysipelas, and on Erythematous Affections in general; as also on the Measles; in which new modes of treatment are communicated. London, Miller, 1802.

Mr. W. considers this ointment as the "very best single application," but finds it necessary occasionally to have recourse to other stimulants, as aromatic vinegar, and the solutions of nitrate of silver and corrosive sublimate.

For the purpose of correcting disordered states of the digestive organs, he is accustomed to give Plummer's pill; and to improve the secretion of the skin itself, he directs the use of Fowler's arsenical solution. In eruptive complaints, he enjoins abstinence from wine, spirits, ale, and porter, shell-fish, dried fish, salmon, herrings, and salt provisions. "Principally," he remarks, "upon this plan, and with these remedies, I have, for many years, succeeded in curing, with comparative ease, the following diseases, viz. prurigo, lepra, psoriasis, pityriasis, impetigo, porrigo, scabies, and sycosis." (P. 38.) And he further asserts, "that, for sixteen years past, in the different varieties of porrigo, I have, with the *same remedies*, and with the *same mode of treatment*, cured, upon the average, 95 cases out of 100, notwithstanding that Dr. Willan says that, "under these varying circumstances, how futile must be the pretensions of those who profess to remove this complaint, and every other form of porrigo, by the same mode of treatment, or by one specific application." "Dr. Bateman, who seems always to be very cautious not to say any thing which his great master has not previously said, makes no remark upon this subject that is worthy of notice." (P. 74.)

Mr. W. has employed his remedies, in a case of LUPUS, with sufficiently good effect, to encourage further trials, (p. 79;) and in another eruption of the face, "which was believed by many of the profession to be lupus," but which he considered to be "only a very exasperated state of psora," much improvement under the same mode of treatment took place.

It does not appear quite clear what disease is intended to be here designated under the name of psora. Previously to Dr. Willan's time the term psora was employed as a generic appellation for the itch; and, by the older writers, the scaly tetter was so denominated. The itch, as the author correctly observes, "avoids the face," (p. 84;) and it seems scarcely probable, that a scaly and superficial affection of the skin, like psoriasis, should, by medical men, be confounded with the tubercular and devastating noli me tangere.

The labours of the celebrated continental dermapathologist are thus described by Mr. Wilkinson. "From the perusal of M. Alibert's inflated fustian, in his 'Description des Maladies de la Peau,' I rise, as I think every sober man must arise, puzzled and disgusted." Surrounded with greater advantages than any other surgeon in Europe, and with Dr. Willan's work open before him, he has neglected that learned author's beautiful arrangement, and has contrived by his perplexing pathology, and by his inefficient and contradictory remedies, to render this department of the science more confused and incomprehensible than he found it. The only remark which he makes upon these diseases, which, in my opinion, is worth our attention, and which might have been expressed in four words, is his conclusion, that *there is nothing like sulphur*." (P. 76.)

The sketch that follows is probably intended for an eminent practitioner of our own country :—

> "Another author, I understand, sits in the professor's chair, laughing at all of us, ridiculing all classification and nomenclature, and all external applications: like David against Goliath, he enters the field against this formidable enemy, armed only with the blue pill and senna tea for his internal action; and for the external, a little hog's-lard, or common cerate, after wiping the eruption particularly dry. Surely this is riding the digestive hobby with a vengeance." P. 76.

---

